# Teaching Basic Life Support to schoolchildren: quasi-experimental study

**DOI:** 10.1590/1518-8345.4078.3340

**Published:** 2020-09-30

**Authors:** Ana Carolina Carraro Tony, Fábio da Costa Carbogim, Daniela de Souza Motta, Kelli Borges dos Santos, Amanda Aparecida Dias, Andyara do Carmo Pinto Coelho Paiva

**Affiliations:** 1 Universidade Federal de Juiz de Fora, Faculdade de Enfermagem, Juiz de Fora, MG, Brazil.

**Keywords:** Nursing, Education, Nursing, Health Education, Cardiopulmonary Resuscitation, Education, Primary and Secondary, Educational Measurement, Enfermagem, Educação em Enfermagem, Educação em Saúde, Reanimação Cardiopulmonar, Ensino Fundamental e Médio, Avaliação Educacional, Enfermería, Educación en Enfermería, Educación en Salud, Reanimación Cardiopulmonar, Educación Primaria y Secundaria, Evaluación Educacional

## Abstract

**Objective:**

to evaluate the knowledge of basic education students before and after educational intervention on Basic Life Support in a situation of adult cardiorespiratory arrest.

**Method:**

quasi-experimental study conducted with 335 students from three elementary schools. Data was collected using an instrument that captured sociodemographic data and knowledge about Basic Life Support. Subsequently, they were analyzed by descriptive and analytical statistics.

**Results:**

students’ knowledge in the post-test (p <0.05) was significantly higher than in the pre-test. The average of the pre-test scores was 4.12 ± 1.7 and, in the post-test it was 6.53 ± 1.9 (p = 0.00).

**Conclusion:**

the results demonstrated effectiveness of the intervention with the expansion of knowledge about Basic Life Support in cardiorespiratory arrest. The results reinforce the importance of Nursing in health education actions in elementary schools.

## Abstract (pt)

**Objetivo:**

avaliar o conhecimento de estudantes do ensino básico antes e após intervenção educativa sobre Suporte Básico de Vida em situação de parada cardiorrespiratória em adultos.

**Método:**

estudo quase-experimental realizado com 335 estudantes de três escolas do ensino básico. Os dados foram coletados por instrumento que captava dados sociodemográficos e conhecimentos sobre Suporte Básico de Vida. Posteriormente, foram analisados por estatística descritiva e analítica.

**Resultados:**

o conhecimento dos estudantes no pós-teste (p < 0,05) foi significativamente maior que no pré-teste. A média das notas no pré-teste foi 4,12 ± 1,7 e, no pós-teste foi 6,53 ± 1,9 (p = 0,00).

**Conclusão:**

os resultados demonstraram a efetividade da intervenção com a ampliação do conhecimento sobre Suporte Básico de Vida em parada cardiorrespiratória e reforçam a importância da Enfermagem nas ações de educação em saúde nas escolas de ensino básico.

## Abstract (es)

**Objetivo:**

evaluar el conocimiento de estudiantes de la enseñanza básica antes y después de la intervención educativa sobre Soporte Básico de Vida, en situación de paro cardíaco en adultos.

**Método:**

estudio casi experimental realizado en 335 estudiantes de tres escuelas de enseñanza básica. Los datos fueron recogidos por un instrumento que captaba datos sociodemográficos y conocimientos sobre Soporte Básico de Vida; posteriormente, estos fueron analizados con la estadística descriptiva y analítica.

**Resultados:**

el conocimiento de los estudiantes en el postest (p < 0,05) fue significativamente mayor que en el pretest. La media de las notas en el pretest fue 4,12 ± 1,7 y en el postest fue 6,53 ± 1,9 (p = 0,00)

**Conclusión:**

los resultados demostraron efectividad de la intervención con la ampliación del conocimiento sobre Soporte Básico de Vida en paro cardíaco y refuerzan la importancia de la Enfermería en las acciones de educación en salud en las escuelas de enseñanza básica.

## Introduction

Basic Life Support (BLS) conducts are defined as initial actions offered by trained people^(^ 1 ^)^to victims of sudden illness, accidents and/or at imminent risk of death. Most of these measures are carried out at the event site until more complex procedures are implemented by health professionals^(^ 1 ^-^ 3 ^)^.

Studies^(^ 1 ^,^ 4 ^)^ pointed out that the main cause of prehospital death is the lack of care, followed by inadequate assistance. In the case of Cardiorespiratory Arrest (CRA), if the resuscitation maneuver is performed within the first minute, the chances of success are up to 98%. From the fifth minute on, the chances drop to 25% and survival rates drop to 1%, if the resuscitation maneuver is performed after ten minutes.

In this sense, the actions provided by trained people are crucial for better prognosis and survival in situations such as hemorrhages, fainting, choking and CRA that occur, in general, outside health institutions^(^ 3 ^)^. Studies have highlighted that the CRA resuscitation maneuver, initiated by trained people until the arrival of emergency services professionals, is associated with a survival rate up to three times higher when compared to victims in CRA who did not receive assistance^(^ 4 ^-^ 6 ^)^.

Cardiovascular diseases are responsible for approximately 15.2 million deaths *per* year worldwide^(^ 7 ^-^ 8 ^)^. Among the countries with the highest mortality rate, Brazil occupies the tenth position, representing 27.7% of deaths, mostly preventable^(^ 7 ^)^, due to heart disease. These data reinforce the need for training to the general population, with the school being an environment conducive to teaching and spreading knowledge about management in situations that require first aid, since children and adolescents are always willing to acquire new knowledge^(^ 2 ^)^.

In the basic education environment, some research has shown that the knowledge and skills of teachers and students in caring for victims in situations of CRA can be improved after educational intervention^(^ 4 ^,^ 9 ^)^. When the offer of educational activities on CRA is regular, the chances of promptness and effectiveness in attendance are greater, considering that knowledge and skills may be reduced over time^(^ 2 ^)^. Therefore, the nurse through the guidelines of the Health at School Program (HSP), figures as one of the main mediators between education and health, being able to act, strategically, in the provision of educational activities in first aid^(^ 10 ^)^.

A study highlighted that, among health professionals, nurses are effective in activities with basic education, as they have been protagonists in the connection between school and health, which enables the acquisition of skills and the establishment of bonds^(^ 11 ^)^. In addition, this professional has managed to extrapolate technical rationality, from a dialogical and reflective practice, in order to enable greater involvement of those who learn^(^ 12 ^)^.

Through the literature, it is evident that, in educational activities aimed at teaching maneuvers in situations of CRA, the nurse instructor must use resources that stimulate the cognitive and metacognitive skills of the apprentice in the field of decision making^(^ 9 ^,^ 13 ^-^ 14 ^)^. Thus, in addition to theoretical information, it is important for nurses to stimulate knowledge and skills through practical pedagogical strategies, with repetition actions, so that the student stops being a mere spectator and starts to act safely^(^ 9 ^)^.

In this sense, the objective of the study was to evaluate the knowledge of basic education students before and after educational intervention on Basic Life Support (BLS) in a situation of adult CRA.

## Method

This is a quasi-experimental study, of the before and after type, non-randomized, in which the experimental group was its own control based on prior knowledge about BLS in a situation of CRA. The research was carried out with elementary school students from three schools in the city of Juiz de Fora, in the State of Minas Gerais, Brazil. These were selected for convenience, with the prerequisite of not having yet adhered to the annual training in First Aid (FA), being under municipal administration and offering the second segment of Elementary Education (6^th^-9^th^ grades), in the morning and afternoon. The sequence of application of the intervention followed the order of acceptance to participate in the research.

At the time of the investigation, the selected schools had 640 students who met the inclusion criteria of being enrolled between the sixth and ninth years of elementary school. The cutoff was established between the sixth and ninth years, since most students would be between 11 and 15 years old, age recommended in the literature as effective for the first approach in theoretical and practical BLS activities^(^ 4 ^)^. As exclusion criteria, we considered those who were away from school activities due to sick leave at the time of data collection.

For the sample calculation, the following formula was used: n= N.Z².p.(1-p)/Z².p.(1-p). + e².(N-1) where: “n” is the calculated sample; “N” is the population; “Z”, the standardized normal variable associated with the confidence level; “P”, the true probability of the event and “e”, the sampling error, using a sampling error of 5% and a 95% confidence level. A sample size of 241 students was obtained. However, the authors preferred to exceed the minimum sample size, 335 students taking part in the investigation.

The selection was made out of convenience according to the students’ availability to participate in the study. Randomization was not performed because it was a study before and after (of the intervention). It is worth mentioning that, in addition to the 335 students, seven others participated in educational activities, but not in the research, either by choice or by non-consent of those responsible.

Data was collected using an instrument previously developed and validated in Brazil^(^ 14 ^)^, capturing sociodemographic information and FA knowledge about BLS in CRA, with closed questions (without punctuation) and multiple choice (with punctuation). The instrument has 13 questions, with questions ranging from the perception of safety in providing care to a victim, to options about performing maneuvers in first aid situations.

For this study, the 13 questions of the questionnaire were evaluated, three of them investigating the previous contact with the theme (without specific score) and ten exclusively with approach to CRA. For each of the ten scoring questions, there are four answer options with only one correct option. The value of each question is one point; so the maximum score is ten and the minimum is zero.

Prior to the educational intervention, the researchers, with authorization from the school management, were in the classrooms, explained about the activities and, for those interested, terms of consent, consent and authorization from those responsible were given. It should be noted that, regardless of the acceptance to participate in the research, the integration of all students with educational activities was guaranteed.

After complying with all ethical and legal procedures, the researchers, along with the school management, drew up a schedule, dividing the students’ training into shifts, classes and schedules. The educational intervention and data collection took place between April and May 2019. The training was conducted for groups of up to 30 students divided into five small groups of activities or stations. Each of these stations received, at random, about six students. This process was repeated until all students received complete training.

In the first station, a pre-test on BLS knowledge was applied to a victim in CRA. This place was reserved with individual seats and tables: each student received a test notebook and pen, without the possibility of discussing the questions, and it was up to him to choose the answer he considered most appropriate. In the second season, a tutor (trained researcher) gave theoretical explanations about the care for an adult victim in a possible cardiac arrest, according to the American Heart Association protocol^(^ 8 ^,^ 15 ^)^.

At the third station, the group, with the help of one of the tutors, performed the practical approach, providing care to a likely victim in CRA. For this, three simulation mannequins were used. In a fourth season, students individually performed the maneuver on a mannequin and, at the end, received feedback on their action, with the possibility of repeating the procedure.

The post-test was performed in the fifth season. In a reserved environment, as in the first season, the student received a new test notebook with the same questions, which should be answered individually, without discussion, based on the knowledge acquired in the educational intervention.

Time was established to carry out the activities in each station: 20 minutes, in each one, for the first and fifth; 15 minutes for the second and 30 minutes, in each one, for the third and fourth, totaling 115 minutes of activity *per* group. To carry out the activities, a tutor was allocated at stations one, two and five, while stations three and four received three tutors each.

For the general evaluation of the answers obtained through the questionnaire, all questions were corrected according to the template present in the instrument used for data collection. Data was analyzed using the statistical program Statistical Package for the Social Sciences (SPSS), version 25.

In the statistical analysis, categorical variables were expressed by means of absolute and relative frequencies and quantitative variables by means and standard deviations. The assumptions of normality of the data were evaluated by the Shapiro-Wilk test. To compare the performance between pre and post-test, by the number of errors and correct answers per question, the chi-square test was used (X²). To assess the students’ average score (in both moments), the paired t-test or the Wilcoxon test was used, according to the distribution of the variables. The standard significance level was set at 0.05 p-value and 95% confidence interval.

The research was initiated after approval by the Ethics Committee in Research with Human Beings of the Federal University of Juiz de Fora under Certificate of Presentation for Ethical Appreciation (CAAE) 90444318.7.0000.5147 and opinion 2,754,970.

## Results

335 students participated in the study, most of them women (n = 202; 60.3%). The mean age of the students was 13.2 ± 1.2 years. Regarding color/race, individuals declared themselves as brown (n= 126; 37.6%), white (n= 115; 34.3%), black (n= 92; 27.5%) and yellow (n= 2; 0.6%). Regarding the series they were attending, most were in the sixth year (n = 105; 31.4%), followed by the seventh (n= 96; 28.6%), eighth (n= 68; 20.3%) and ninth (n= 66; 19.7%). There was no significant association (p> 0.05) between the knowledge of BLS to a victim in CRA and the variables age and sex.

When asked about previous knowledge about BLS, the majority (n= 294; 87.8%) reported never having participated in courses or training; however, 189 (56.4%) had already witnessed situations that required provision of First Aid (FA). Among the situations, those with a clinical condition recognized by the students stood out (n= 141; 42.1%), followed by clinical emergencies, such as hypotension, syncope and seizure crisis (n= 63; 18.18%) and heart attack (n= 49; 14.6%). Regarding the conduct established in the face of the situation, the majority (n= 212; 63.3%) did not know what to do, because they did not feel safe. Another 123 (36.7%) performed some action that they considered correct, such as checking a pulse, sitting down or giving water to the person, and then took the victim to the hospital.

Table 1 shows, comparatively, the frequency and significance of students’ responses in the pre and post-test on BLS to a victim in CRA.

**Table 1:** Evaluation of Elementary School students (n = 335) regarding performance in the pre and post-test. Juiz de Fora, MG, Brazil, 2019

Questions	Pre-test		Post-test		p
	n	%	n	%	
Do you know how to check vital signs?					
Wrong	127	38.0	55	16.4	0.000
Right	208	62.0	280	83.6	
Which measure is taken when someone is unconscious?					
Wrong	274	81.8	223	66.6	0.000
Right	61	18.2	112	33.4	
Why is a correct provision of FA necessary*?					
Wrong	108	32.2	53	15.8	0.000
Right	227	67.8	282	84.2	
How do you check if the victim is breathing?					
Wrong	34	10.1	16	4.8	0.012
Right	301	89.9	319	95.2	
Would you do cardiac massage / mouth to mouth resuscitation?					
Wrong	175	52.2	74	22.1	0.000
Right	160	47.8	261	77.9	
What is cardiac massage?					
Wrong	265	79.1	156	46.6	0.000
Right	70	20.9	179	53.4	
What is the purpose of cardiac massage?					
Wrong	229	68.4	196	58.5	0.016
Right	106	31.6	139	41.5	
What is the victim's position for CRA care^†^?					
Wrong	173	51.6	132	39.4	0.001
Right	162	48.4	203	60.6	
Is it the proper body location to perform the massage?					
Wrong	250	74.6	114	34.0	0.000
Right	85	25.4	221	66.0	
Do you know how many times per minute you should perform the massage?					
Wrong	320	95.5	126	37.6	0.000
Right	15	4.5	209	62.4	

* FA = First aid;

† CRA = Cardio respiratory arrest

From the data presented in Table 1, it is possible to infer that the students’ knowledge in the post-test (p <0.05) was significantly higher than in the pre-test. The average of the pre-test scores was 4.12 ± 1.7 and, in the post-test, the average was 6.53 ± 1.9 (p= 0.00).

**Figure 1:**
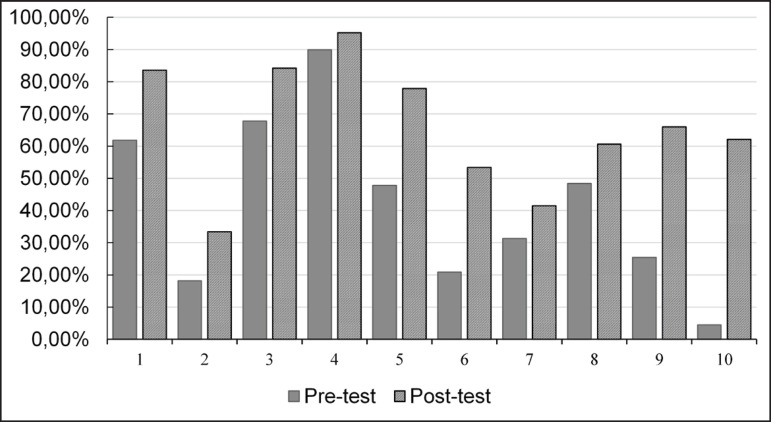
Assertive index *per* question before and after educational intervention with Elementary School students (n= 335). Juiz de Fora, MG, Brazil, 2019

It can be confirmed, by exploring the data in Figure 1, that, in all questions, the rate of correct answers after the theoretical-practical exposure was higher than the previous one.

## Discussion

The school is understood as a multidimensional space for sharing relationships, culture, stories and knowledge. One of the aspects desirably included in the school environment is health education mediated by universities, services and health professionals. Studies^(^ 8 ^,^ 16 ^-^ 17 ^)^ highlighted the central role of the professional nurse in educational practices in basic education, be they health promotion or disease prevention. This role is based mainly on sexual education, hygiene, food, immunization and mental health^(^ 8 ^,^ 17 ^-^ 18 ^)^.

On the other hand, the teaching of FA at school, with emphasis on approaches in situations of CRA, has received international attention and prominence in the nursing literature^(^ 4 ^,^ 19 ^-^ 21 ^)^, despite the scarcity of studies that address the theme with elementary school students.

In Brazil, as of 2017, annual training in FA has become mandatory for professionals from public and private schools, however, the educational approach aimed at students is still optional^(^ 2 ^-^ 3 ^)^. The researches^(^ 4 ^,^ 19 ^-^ 21 ^)^ teaching BLS to a victim in cardiac arrest has been carried out, mostly, with students aged between 11 and 15 years, with no direct implications of gender and age on results or performance. These data is in line with the sociodemographic findings of this investigation.

On the other hand, a study carried out by nurses from Jordan demonstrated that the success in the theoretical-practical activities of BLS was linked to the older age of students in the second segment of Elementary Education^(^ 22 ^)^.

Nevertheless, a comparative investigation between the performance of students from Brazilian public and private schools identified performance and superior knowledge retention in the second group, although the authors detected a significant increase (p<0.005) of knowledge in both groups^(^ 21 ^)^.

In this research, it was found that the majority of students had never participated in training on the subject and did not feel safe to provide the correct FA actions. Although there is no consensus in the literature on the appropriate time to learn about this topic, some studies highlight that the teaching of FA has happened late, including to undergraduate nursing students^(^ 4 ^,^ 23 ^-^ 25 ^)^. Studies^(^ 20 ^-^ 23 ^)^ evidenced, through specific tests, that there is a significant increase in safety and willingness to provide BLS assistance in situations of CRA after theoretical-practical training of students or the general population.

In a research^(^ 26 ^)^ held in Saudi Arabia, with a population of 508 high school students, low prior knowledge was identified, in addition to low security and willingness to assist in CRA. However, after the theoretical-practical educational intervention, there was a significant improvement in knowledge and skills. These data corroborate the findings of this study, in which there was a significant variation between the pre and immediate post-test, demonstrating an improvement in the knowledge acquired.

It should be noted, however, that there are evidences that the retention of knowledge and skills in cardiopulmonary resuscitation maneuvers declines over time and updates are recommended^(^ 5 ^,^ 8 ^,^ 21 ^)^. Nursing professionals can contribute with updates and training on FA and BLS to professionals from elementary schools and their students^(^ 27 ^-^ 28 ^)^. Studies^(^ 21 ^,^ 28 ^)^ pointed out that well-trained individuals can become potential disseminators of knowledge to family and friends, expanding the network of trained people.

With the results of this research, advances in Nursing knowledge within the scope of basic education, as it is clear that there is a lack of studies on BLS care in situations of CRA in this environment. The study fosters scientific knowledge in Nursing and signals the need for advances in research on teaching BLS to students. As a limitation, it is noteworthy that there was no late post-test, evaluating the knowledge retention of students in the medium and long terms.

## Conclusion

Through an educational intervention, the study sought to assess the knowledge of basic education students on Basic Life Support in situations of cardiorespiratory arrest. In this sense, the results demonstrated the effectiveness of the intervention with the expansion of the knowledge acquired through the comparison between the pre and post-test. The results reinforce the importance of Nursing in health education actions in elementary schools.
